# Steady state imaging of the thoracic vasculature using inversion recovery FLASH and SSFP with a blood pool contrast agent

**DOI:** 10.1186/1532-429X-14-S1-P51

**Published:** 2012-02-01

**Authors:** Mauricio S Galizia, Jennifer A Febbo, Andrada R Popescu, Xiaoming Bi, Jeremy Collins, Michael Markl, Robert R Edelman, James Carr

**Affiliations:** 1 Radiology, Northwestern University, Chicago, IL, USA; 2 Radiology, NorthShore University HealthSystem, Evanston, IL, USA; 3 Medical Imaging, Children's Memorial Hospital, Chicago, IL, USA; 4 Cardiovascular MR R&D, Siemens Healthcare, Chicago, IL, USA

## Background

Although contrast-enhanced first-pass MRA (FP-MRA) is frequently used to visualize the thoracic vasculature, it may not be ideal for the assessment of the aortic root due to poor image quality and motion artifact. Blood-pool contrast agents remain within the intravascular space for several hours, allowing vessels to be imaged longer and therefore improving spatial resolution. The purpose of this study is to compare steady-state magnetic resonance angiography (SS-MRA) following injection of a blood-pool contras agent to first-pass MR angiography (FP-MRA) in adults with thoracic aortic disease.

## Methods

25 patients (14 men, 11 women) with suspect thoracic aortic disease disease underwent MRA on a 1.5 T scanner (Magnetom Aera and Avanto; Siemens Medical Solutions). The MRA protocol consisted of FP-MRA followed by SS-MRA after intravenous injection of gadofosveset trisodium (Ablavar, Lantheus Medical Imaging). FP-MRA consisted of a breath-held ECG-gated FLASH acquisition in a sagittal oblique orientation with the following imaging parameters: TR/TE: 2.8/1.0, flip angle 25°, FOV 343x500 mm, matrix 264x512, slice thickness 1.5 mm, voxel size 1.3 x 1.0 x 1.0 mm, GRAPPA x 2, 20 second acquisition. 0.03 mmol/kg of gadofosveset was injected intravenously at 1cc/sec in an antecubital vein. Contrast bolus timing was achieved using care bolus technique. SS-MRA consisted of free-breathing ECG-gated IR-FLASH and IR-SSFP in a sagittal oblique orientation. IR-FLASH had the following parameters: TR/TE/TI: 3.5/1.5/260, flip angle 18°, and IR-SSFP had: TR/TE/TI: 3.3/1.5/260, flip angle 70°. Both sequences had FOV 326x380, matrix 440x512, slice thickness 1.5 mm, voxel 0.7 x 0.7 x 1.0 mm, GRAPPA x 2, and 3 minute acquisition. Respiratory gating was achieved using a navigator acquisition with an average acceptance window of 35%. For quantitative analysis, orthogonal dimensions of the thoracic aorta were measured at several locations. Signal-to-noise ratio (SNR) was also measured for both techniques by placing regions of interest in the aortic root and the ascending aorta. For qualitative analysis, two independent reviewers evaluated both FP-MRA and SS-MRA images separately. The aortic root and the ascending aorta were scored on an image quality scale of 1-4.

## Results

There was no significant difference in aortic dimensions at all anatomic locations between FP-MRA and SS-MRA. SNR was higher for SS-MRA compared to FP-MRA. Image quality scores were higher for SS-MRA compared to FP-MRA. Thoracic aortic aneurysms were detected equally between both techniques.

## Conclusions

SS-MRA following injection of blood pool contrast agent was comparable to FP-MRA for measurement of aortic dimensions. SNR and image quality was higher with SS-MRA. SS-MRA may be a useful adjunct to FP-MRA in cases of bolus mistiming, or may potentially replace FP-MRA, thereby simplifying MRA assessment of the thoracic vasculature.

## Funding

None.

**Figure 1:**
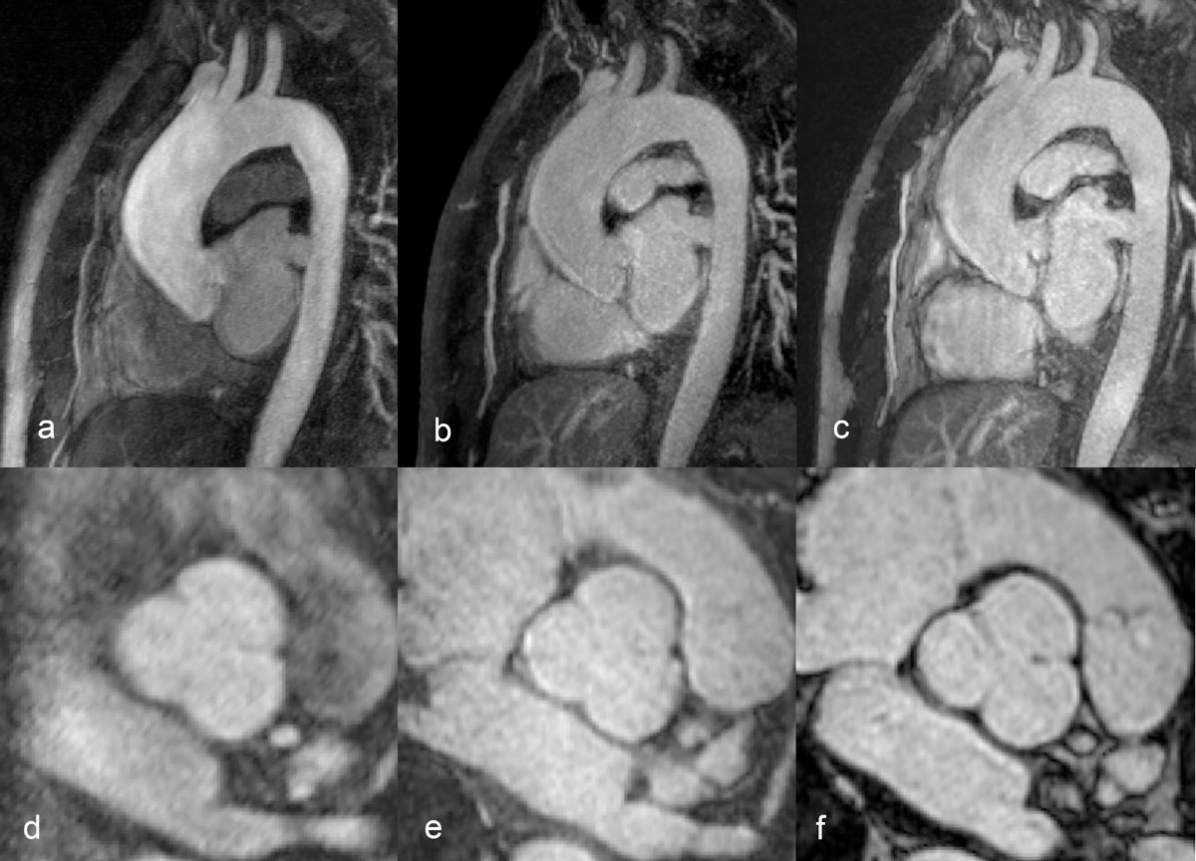
MRA of the thoracic aorta of a 57-year-old man in a sagittal oblique orientation (a-c) and in a multiplane reformat image (MPR) orthogonal to the sinus of Valsalva (d-f). The image quality of FP-MRA (a, d) is not as high as in the SS-MRA images: IR-FLASH (b, e) and IR-SSFP (c, f).

